# The effect of emergency surgery on acute abdomen patients with COVID-19 pneumonia: a retrospective observational study

**DOI:** 10.18632/aging.103839

**Published:** 2020-08-15

**Authors:** Ning Zhao, Liang Wu, Yifeng Cheng, Hai Zheng, Ping Hu, Chaojie Hu, Ding Chen, Peng Xu, Qingyong Chen, Ping Cheng, Jinhuang Chen, Gang Zhao

**Affiliations:** 1 Department of Gastrointestinal Surgery, Union Hospital, Tongji Medical College, Huazhong University of Science and Technology, Wuhan 430022, China; 2 Department of Gastrointestinal Surgery, Union Hospital West Campus, Tongji Medical College, Huazhong University of Science and Technology, Wuhan 430056, China; 3 Department of Emergency Surgery, Union Hospital, Tongji Medical College, Huazhong University of Science and Technology, Wuhan 430022, China

**Keywords:** COVID-19, acute abdomen, emergency surgery

## Abstract

During the COVID-19 outbreak, some patients with COVID-19 pneumonia also suffered from acute abdomen requiring surgical treatment; however, there is no consensus for the treatment of such patients. In this study, we retrospectively reviewed 34 patients with acute abdomen who underwent emergency surgery during the COVID-19 outbreak. Among the 34 patients with acute abdomen, a total of six cases were found with COVID-19 pneumonia (clinical classification for COVID-19 pneumonia: all were the common type). On the premise of similar demographics between both groups, patients with COVID-19 pneumonia had worse indicators of liver and coagulation function. Compared with acute abdomen patients without COVID-19, patients with COVID-19 pneumonia had a longer hospital stay, but there were no significant differences in postsurgical complications (P = 0.58) or clinical outcomes (P = 0.56). In addition, an obvious resolution of lung inflammation after surgery was observed in five COVID-19 patients (83.3%). No new COVID-19 cases occurred during the patients’ hospital stays. Therefore, for the common type of COVID-19 pneumonia, emergency surgery could not only improve the outcomes of COVID-19 pneumonia patients with acute abdomen, but also benefit the resolution of pulmonary inflammation.

## INTRODUCTION

In the last two decades there have been two large-scale pandemics caused by coronaviruses, severe acute respiratory syndrome (SARS) [1] and Middle East respiratory syndrome (MERS) [2]. At the end of 2019, another novel coronavirus, designated as severe acute respiratory syndrome coronavirus 2 (SARS-CoV-2), emerged in Wuhan and subsequently spread rapidly throughout the world [3, 4]. Due to accumulating evidence of continuous person-to-person transmission and a general susceptibility of humans to the virus [5–7], the World Health Organization (WHO) declared coronavirus disease 2019 (COVID-19) a public health emergency of international concern on January 30, 2020. As of May 16, 2020, COVID-19 caused 309,713 deaths among over 4.5 million patients across more than 200 countries, with a case-fatality rate of 6.8%. Although SARS-CoV-2 was found to predominantly infect the lower airways and cause life-threatening pneumonia [8, 9], evidence has revealed that the digestive system might be another potential viral target [7, 10, 11].

Acute abdomen is defined as acute onset of abdominal pain which requires accurate diagnosis and treatment within a particular time limit to prevent mortality and morbidity [12]. During COVID-19 outbreaks, some patients with COVID-19 pneumonia also suffered from acute abdomen requiring immediate interventions [13]. However, previous studies have demonstrated that preoperative pneumonia is a significant risk factor for poor postsurgical outcomes [14, 15]. In addition, surgical treatment might increase medical staff exposure to SARS-CoV-2 [16, 17] and trigger excessive inflammation in the patient, resulting in worsening of COVID-19 pneumonia [18]. Therefore, an investigation of the impact of emergency surgery on patients with both acute abdomen and COVID-19 pneumonia is urgently needed.

## RESULTS

### Clinical characteristics of patients

Among the 34 patients with acute abdomen who underwent emergency surgery, six patients had COVID-19, and the remaining 28 patients did not. The baseline characteristics of all patients are summarized in Table 1. No new infections were found in medical staff or patients throughout the hospitalization period.

**Table 1:** The baseline characteristics of all patients with acute abdomen.

**Characteristics**	**Patients with acute abdomen**			**P-value**
	**With COVID-19 (n = 6)**		**Without COVID-19 (n = 28)**	
Age (years)	70 ± 4.2		55 ± 22	0.120
Gender				0.170
Female	4 (67%)		9 (32%)	
Male	2 (33%)		19 (68%)	
Diagnosis				0.060
Acute appendicitis	2 (33%)		12 (43%)	
Gastrointestinal perforation	0 (0%)		10 (36%)	
Intestinal obstruction	3 (50%)		5 (18%)	
Gangrenous cholecystitis	1 (17%)		0 (0%)	
Bladder rupture	0 (0%)		1 (4%)	
Comorbidities				0.670
No	3 (50%)		11 (39%)	
Yes	3 (50%)		17 (61%)	
Laboratory findings				
WBC (×10^9^/L)	10.4 ± 6.5		11.8 ± 3.8	0.490
Neutrophil(×10^9^/L)	8.9 ± 5.9		10.1 ± 3.5	0.510
Lymphocyte (×10^9^/L)	0.7 ± 0.3		1.1 ± 0.7	0.260
HGB (g/L)	107.2 ± 26.8		143.9 ± 17.4	<0.001
CRP (mg/L)	82.6 ± 72.9		139.2 ± 67.1	0.074
PCT (μg/L)	3.4 ± 5.3		8.8 ± 8.7	0.160
Albumin (g/L)	30 ± 10.8		41.6 ± 6.5	0.001
ALT (U/L)	70.7 ± 108.3		18.7 ± 7	0.012
AST (U/L)	72.7 ± 93.7		20.6 ± 13.7	0.006
D-Dimer (mg/L)	2.6 ± 3.3		1.4 ± 1.2	0.140
APTT (s)	50.7 ± 10		36.1 ± 3.6	<0.001
PT (s)	16.9 ± 4.5		14.1 ± 1.2	0.006

Abbreviations: ALT, alanine aminotransferase; AST, aspartate aminotransferase; APTT, activated partial thromboplastin time; PT, prothrombin time; HGB, hemoglobin; WBC, white blood cell; CRP, C-reaction protein; PCT, procalcitonin.

**Figure 1:**
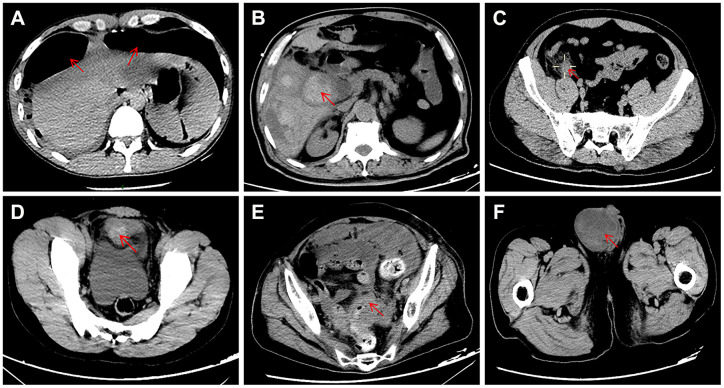
**Typical appearance of abdominal CT showing the causes of acute abdomen in the present study.** (**A**) duodenal perforation accompanied by free intraperitoneal gas; (**B**) gangrenous cholecystitis; (**C**) acute appendicitis; (**D**) bladder rupture; (**E**) intestinal obstruction caused by carcinomas in the rectosigmoid junction; (**F**) intestinal obstruction caused by inguinal incarcerated hernia.

Of the 28 patients who did not have COVID-19 pneumonia (9 female and 19 male; mean age 55 years (range 17–87)), 12 (43%) patients were diagnosed with acute appendicitis, 10 (36%) with gastrointestinal perforation, 5 (18%) with intestinal obstruction and 1 (4%) with bladder rupture. The typical abdominal CT appearance is shown in Figure 1. Comorbidities were found in 17 (61%) patients and included diabetes mellitus in 7 (25%), coronary heart disease in 7 (25%), hypertension in 6 (21.4%), chronic obstructive pulmonary disease in 2 (7.1%), chronic renal failure in 1 (3.6%), chronic liver failure in 1 (3.6%), acute myeloid leukemia in 1 (3.6%), and rheumatoid arthritis in 1 (3.6%). Five (17.9%) patients were reported to have postoperative complications: one had intra-abdominal infection, one had a wound infection, and three had multiple organ dysfunction syndrome (MODS). All three patients with postoperative MODS had preoperative comorbidities (case 1: coronary heart disease and chronic renal failure; case 2: chronic liver failure; case 3: hypertension and coronary heart disease). In total, 25 (89.3%) patients were cured, and the remaining 3 patients died due to severe septic shock and MODS.

The detailed clinical characteristics of the six patients with both acute abdomen and COVID-19 pneumonia are shown in Table 2. Age of the six patients (4 women and 2 men) ranged from 66 to 78 years. Three patients were diagnosed with intestinal obstruction, two with acute appendicitis, and one with gangrenous cholecystitis. The clinical classification of COVID-19 pneumonia in all patients was the common type. Three patients had hypertension, and one had coronary heart disease. The most common clinical manifestations were abdominal pain and fever. Two (33.3%) patients tested positive for SARS-CoV-2 by RT-PCR, and one patient tested positive for IgM-IgG antibodies; however, typical CT imaging manifestations of COVID-19 pneumonia were found in all six patients. Postoperative complications occurred in two patients: one had aspiration pneumonia and the other had MODS. All patients received antiviral therapy (ribavirin, 500 mg each time, twice times a day, 5-7 days; arbidol, 200 mg each time, three times a day, 5-7 days; Interferon α-2b, 5.0×10^5^ IU, nebulized inhalation, twice times a day) and antibacterial therapy, and four patients received immunoglobulins (human immunoglobulin, 10g/d). Two patients with postoperative complications received mechanical ventilation and systematic corticosteroid treatment (methylprednisolone, 1–2 mg/kg.d, 3–5 days). In total, five patients were cured, and one patient died of postoperative MODS.

**Table 2:** The clinical characteristics of the six patients with both acute abdomen and COVID-19 pneumonia.

**Characteristics**	**Case 1**	**Case 2**	**Case 3**	**Case 4**	**Case 5**	**Case 6**
Age, years	69	78	68	68	66	69
Gender	Female	Male	Female	Male	Female	Female
Evidence of COVID-19						
RT-PCR	Negative	Positive	Negative	Negative	Negative	Positive
IgM-IgG antibodies	NA	NA	NA	NA	Negative	Positive
Typical CT manifestation	Unilateral	Bilateral	Bilateral	Unilateral	Bilateral	Bilateral
Diagnosis	Intestinal volvulus Pneumonia (mild)	Gangrenous cholecystitis Pneumonia (mild)	Acute appendicitis Pneumonia (mild)	Malignant intestinal obstruction Pneumonia (mild)	Acute appendicitis Pneumonia (mild)	Malignant intestinal obstruction Pneumonia (mild)
Symptoms and signs						
Fever	No	Yes	Yes	Yes	Yes	No
Cough	Yes	No	No	Yes	No	No
Expectoration	No	Yes	No	Yes	No	Yes
Abdominal pain	Yes	Yes	Yes	Yes	Yes	Yes
Diarrhea	No	No	No	No	Yes	No
Nausea and vomiting	Yes	Yes	No	Yes	No	Yes
Comorbidities	No	Hypertension	No	Hypertension, CHD	Hypertension	No
Postoperative complications	No	MODS	No	Aspiration pneumonia	No	No
Treatment						
Mechanical ventilation	No	Yes	No	Yes	No	No
Antibiotics	Yes	Yes	Yes	Yes	Yes	Yes
Antivirals	Yes	Yes	Yes	Yes	Yes	Yes
Immune globulins	Yes	Yes	No	Yes	Yes	No
Hormones	No	Yes	No	Yes	No	No
Clinical outcome	Discharged	Death	Discharged	Discharged	Discharged	Discharged

Abbreviations: NA, not available; CHD, coronary heart disease; MODS, multiple organ dysfunction syndrome.

### Emergency surgery could not only improve the outcomes of acute abdomen patients with COVID-19 pneumonia, but also benefit the resolution of pulmonary inflammation

The baseline characteristics in patients with and without COVID-19 pneumonia are shown in Table 1. Differences in demographics, including age (P = 0.12), sex (P = 0.17), diagnosis (P = 0.06) and comorbidities (P = 0.67), between both groups were not significant. However, patients with COVID-19 pneumonia had higher ALT (70.7 ± 108.3 U/L vs. 18.7 ± 7 U/L, P = 0.012), AST (72.7 ± 93.7 U/L vs. 20.6 ± 13.7 U/L, P = 0.006), APTT (50.7 ± 10 s vs. 36.1 ± 3.6 s, P < 0.001), and PT (16.9 ± 4.5 s vs. 14.1 ± 1.2 s, P = 0.006), and lower albumin (30 ± 10.8 g/L vs. 41.6 ± 6.5 g/L, P = 0.012) and hemoglobin (107.2 ± 26.8 g/L vs. 143.9 ± 17.4 g/L, P < 0.001) than patients who did not have COVID-19 pneumonia. In addition, although there were no significant differences, patients with COVID-19 pneumonia had lower infection-related biomarkers, including WBC ((10.4 ± 6.5)×10^9^/L vs. (11.8 ± 3.8)×10^9^/L, P = 0.49), lymphocyte ((0.7 ± 0.3)×10^9^/L vs. (1.1 ± 0.7)×10^9^/L, P = 0.26), neutrophil ((8.9 ± 5.9)×10^9^/L vs. (10.1 ± 3.5)×10^9^/L, P = 0.51), CRP (82.6 ± 72.9 mg/L vs. 139.2 ± 67.1 mg/L, P = 0.074) and PCT (3.4 ± 5.3 μg/L vs. 8.8 ± 8.7 μg/L, P = 0.16), than patients who did not have COVID-19 pneumonia.

**Figure 2:**
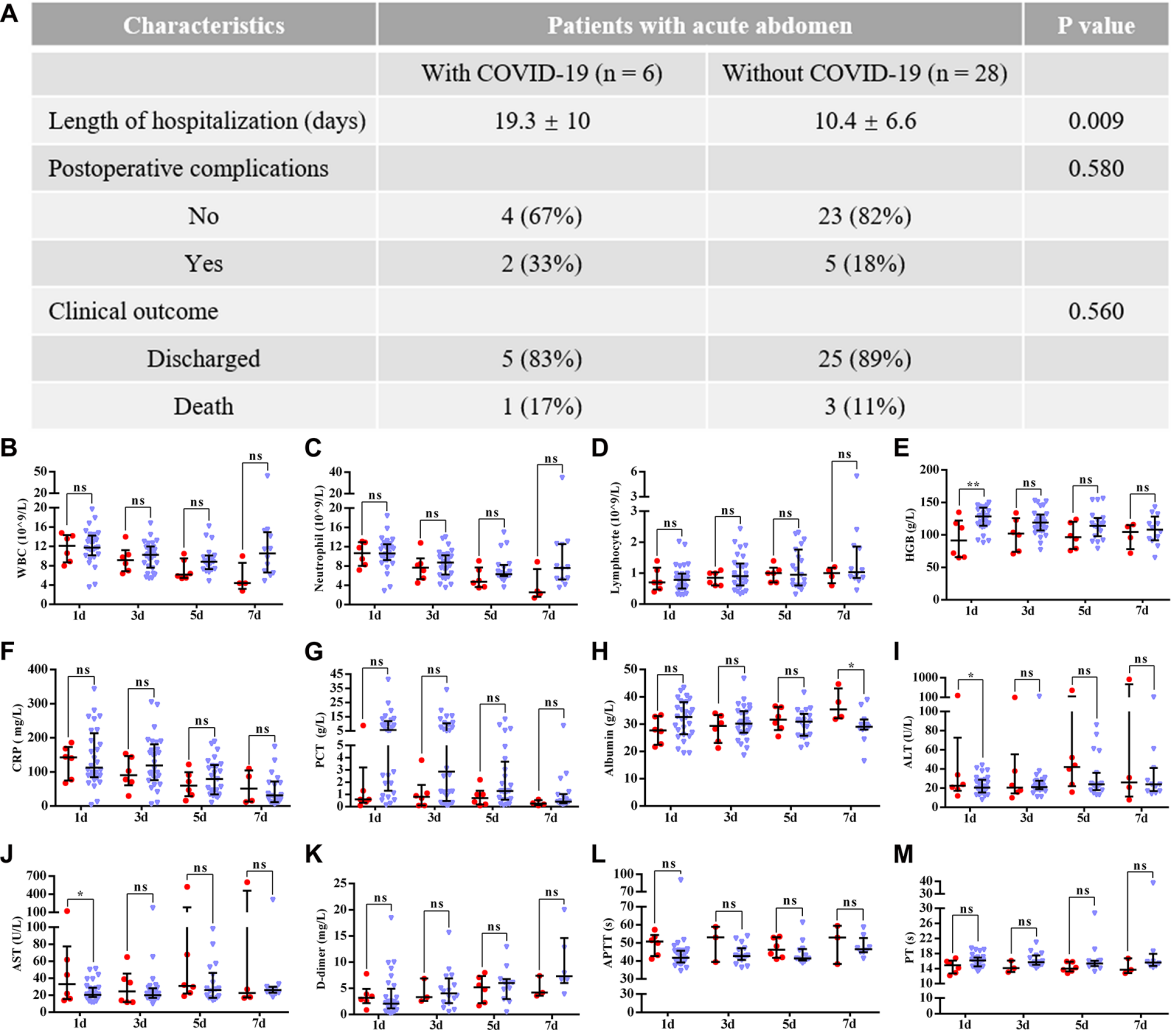
**Postoperative outcomes of all patients with acute abdomen.** Data are presented as numbers and percentages for categorical variables, and continuous data are expressed as the mean ± standard deviation (SD). *P< 0.05, **P< 0.01, ***P <0.001, based on Student’s t-test. (**A**) the difference between both groups in clinical outcomes; (**B**–**M**) shows the differences between patients with and without COVID-19 pneumonia in postoperative laboratory findings, including (**B**) WBCs (white blood cells); (**C**) neutrophils; (**D**) lymphocytes; (**E**) HGB (hemoglobin); (**F**) CRP (C-reactive protein); (**G**) PCT (procalcitonin); (**H**) Albumin; (**I**) ALT (alanine aminotransferase); (**J**) AST (aspartate aminotransferase); (**K**) D-dimer; (**L**) APTT (activated partial thromboplastin time); (**M**) PT (prothrombin time). Red and blue marks represent patients with and without COVID-19 pneumonia, respectively.

**Figure 3:**
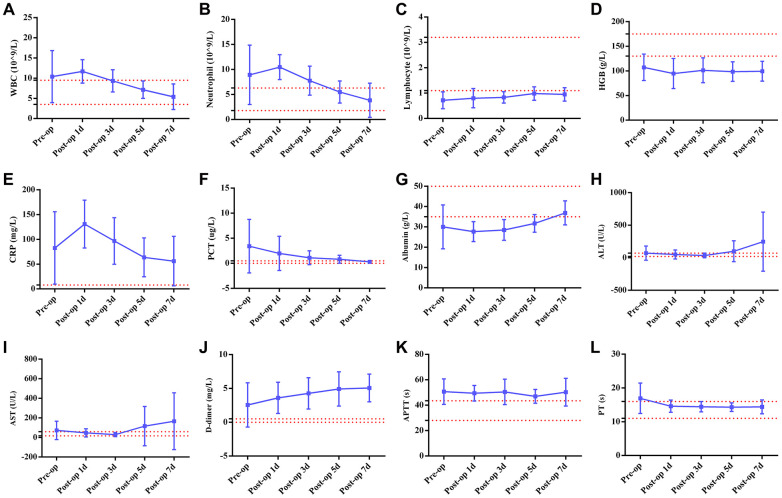
**Line graphs illustrating detailed changes in laboratory findings in six patients with both acute abdomen and COVID-19 pneumonia.** The red line represents the normal range of laboratory findings. (**A**) WBCs (white blood cells); (**B**) neutrophils; (**C**) lymphocytes; (**D**) HGB (hemoglobin); (**E**) CRP (C-reactive protein); (**F**) PCT (procalcitonin); (**G**) Albumin; (**H**) ALT (alanine aminotransferase); (**I**) AST (aspartate aminotransferase); (**J**) D-dimer; (**K**) APTT (activated partial thromboplastin time); (**L**) PT (prothrombin time).

The comparative data of postsurgical outcomes between the two groups are shown in Figure 2. Compared with patients who did not have COVID-19 pneumonia, patients with COVID-19 pneumonia had a longer hospital stay (19.3 ± 10 days vs. 10.4 ± 6.6 days, P = 0.009), but no significant differences in postsurgical complications (P = 0.58) and clinical outcomes (P = 0.56) were found between groups. Furthermore, the majority of worsening preoperative laboratory indicators, including ALT (P = 0.43), AST (P = 0.93), APTT (P = 0.1), PT (P = 0.14), albumin (P = 0.44) and hemoglobin (P = 0.06), had improved by the third postoperative day. As outlined in Figure 3, when compared with preoperative indicators, postoperative infection-related biomarkers also decreased, including WBCs ((10.4 ± 6.5)×10^9^/L vs. (5.4 ± 3.2)×10^9^/L, P = 0.19), neutrophils ((8.9 ± 5.9)×10^9^/L vs. (3.9 ± 3.4)×10^9^/L, P = 0.16), CRP (82.6 ± 72.9 mg/L vs. 56.1 ± 49.8 mg/L, P = 0.55) and PCT (3.4 ± 5.3 μg/L vs. 0.3 ± 0.2 μg/L, P = 0.29).

**Figure 4:**
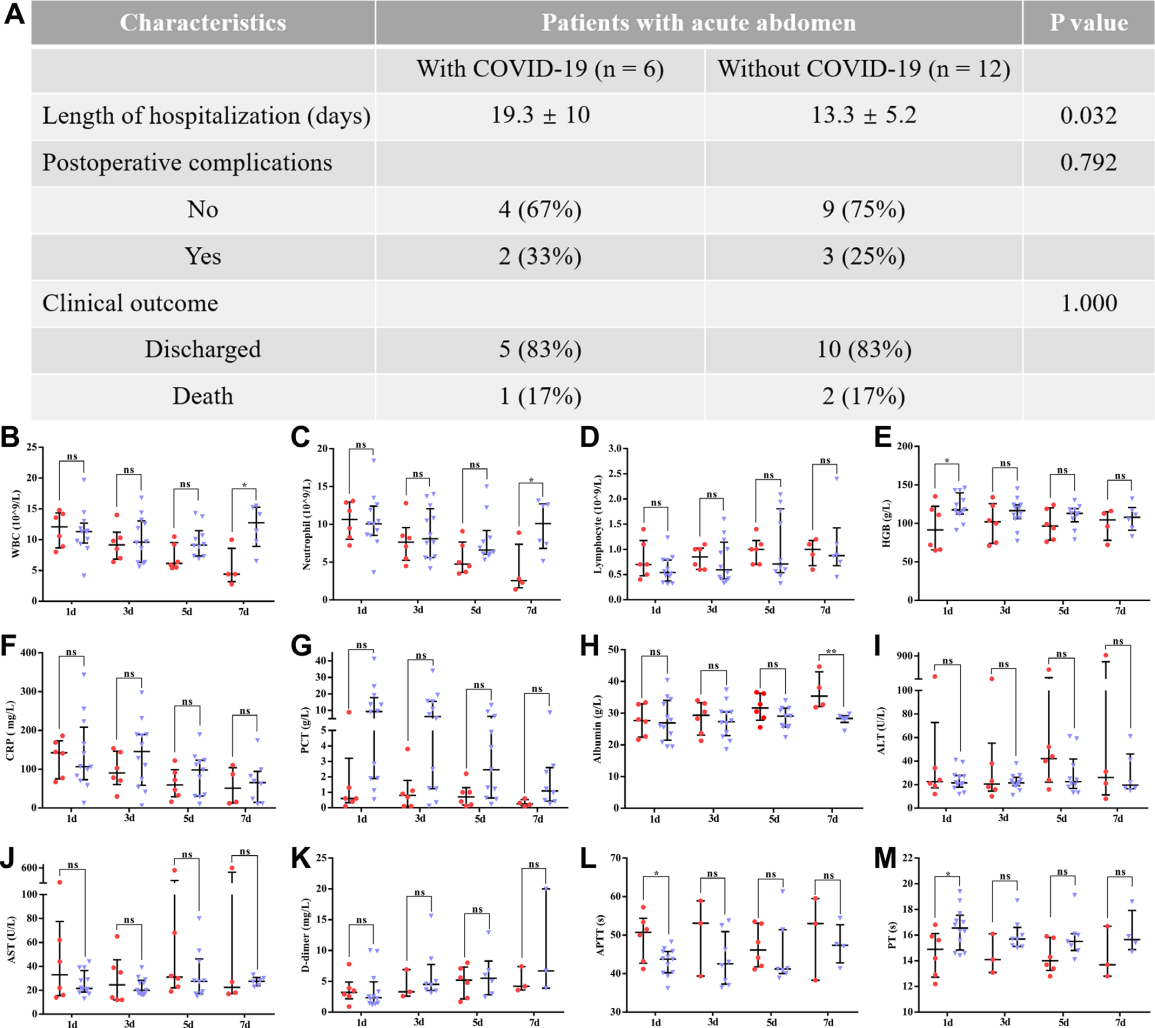
**The difference between patients with COVID-19 pneumonia and those without COVID-19 pneumonia (aged between 60 and 80) in postoperative outcomes.** Data are presented as numbers and percentages for categorical variables, and continuous data are expressed as the mean ± standard deviation (SD). *P< 0.05, **P< 0.01, ***P <0.001, based on Student’s t-test. (**A**) The difference between both groups in clinical outcomes; (**B**–**M**) shows the differences in postoperative laboratory findings between patients with and without COVID-19 pneumonia, including (**B**) WBCs (white blood cells); (**C**) neutrophils; (**D**) lymphocytes; (**E**) HGB (hemoglobin); (**F**) CRP (C-reactive protein); (**G**) PCT (procalcitonin); (**H**) Albumin; (**I**) ALT (alanine aminotransferase); (**J**) AST (aspartate aminotransferase); (**K**) D-dimer; (**L**) APTT (activated partial thromboplastin time); (**M**) PT (prothrombin time). Red and blue marks represent patients with and without COVID-19 pneumonia, respectively.

**Figure 5:**
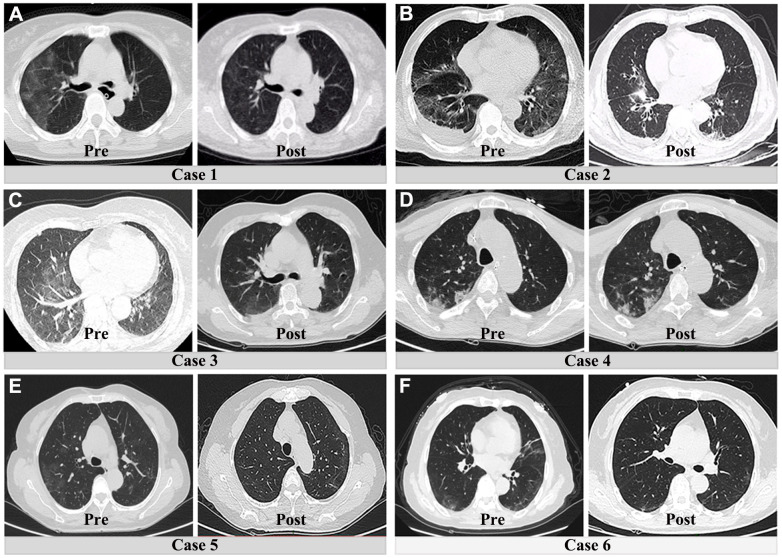
**Preoperative and postoperative CT lung manifestations in six patients with both acute abdomen and COVID-19 pneumonia.** (**A**–**C**) and (**E**, **F**) show the obvious resolution of pulmonary inflammation. The fourth patient had no significant change of pulmonary inflammation after surgical treatment (**D**).

To remove the potential impact of age on the above results, we further compared the pre- and postsurgical differences between patients with COVID-19 and those without COVID-19 pneumonia (between 60 and 80 years old). After age-matching between both groups, the majority of preoperative and postoperative results were consistent with the previous results. As shown in Table 3, patients with COVID-19 pneumonia still had poor preoperative liver and coagulation function. However, the bulk of abnormal preoperative laboratory findings were significantly and rapidly corrected after surgical treatment (Figure 4). In addition, an obvious resolution of lung inflammation was observed after surgery in five patients (83.3%) (Figure 5). These results indicated that COVID-19 pneumonia is associated with poor liver function and coagulation function in acute abdomen patients with COVID-19 pneumonia. Nevertheless, emergency surgery could not only improve the outcomes of COVID-19 pneumonia patients with acute abdomen, but also benefit the resolution of pulmonary inflammation.

**Table 3:** The preoperative differences between patients with COVID-19 pneumonia and those without COVID-19 pneumonia after age-matching.

**Characteristics**	**Patients with acute abdomen**			**P-value**
	**With COVID-19 (n = 6)**		**Without COVID-19 (n = 12)**	
Age (years)	70 ± 4.2		71.2 ± 5.9	0.590
Gender				0.620
Female	4 (67%)		5 (42%)	
Male	2 (33%)		7 (58%)	
Diagnosis				0.110
Acute appendicitis	2 (33%)		5 (42%)	
Gastrointestinal perforation	0 (0%)		5 (42%)	
Intestinal obstruction	3 (50%)		2 (17%)	
Gangrenous cholecystitis	1 (17%)		0 (0%)	
Comorbidities				0.340
No	3 (50%)		3 (25%)	
Yes	3 (50%)		9 (75%)	
Laboratory findings				
WBC (×10^9^/L)	10.4 ± 6.5		9.6 ± 2.6	0.730
Neutrophil(×10^9^/L)	8.9 ± 5.9		8.3 ± 2.4	0.750
Lymphocyte (×10^9^/L)	0.7 ± 0.3		1.0 ± 0.7	0.300
HGB (g/L)	107.2 ± 26.8		143.6 ± 13.2	0.001
CRP (mg/L)	82.6 ± 72.9		148.9 ± 79.7	0.110
PCT (μg/L)	3.4 ± 5.3		11.9 ± 11.5	0.110
Albumin (g/L)	30 ± 10.8		38.3 ± 5.2	0.040
ALT (U/L)	70.7 ± 108.3		15.3 ± 4.8	0.086
AST (U/L)	72.7 ± 93.7		15.8 ± 6.3	0.046
D-Dimer (mg/L)	2.6 ± 3.3		1.4 ± 1.1	0.290
APTT (s)	50.7 ± 10		37.2 ± 4.4	<0.001
PT (s)	16.9 ± 4.5		14.1 ± 0.8	0.042

Abbreviations: ALT, alanine aminotransferase; AST, aspartate aminotransferase; APTT, activated partial thromboplastin time; PT, prothrombin time; HGB, hemoglobin; WBC, white blood cell; CRP, C-reaction protein; PCT, procalcitonin.

## DISCUSSION

Accurate diagnosis and appropriate intervention within a particular time limit is crucial to prevent deterioration and mortality in patients with acute abdomen [12]. Although previous studies revealed that preoperative pneumonia is significantly associated with worse postoperative outcomes [14, 15], there is still no direct evidence suggesting that surgical treatment leads to adverse effects in acute abdomen patients with COVID-19 pneumonia. Using the data from 34 patients with acute abdomen who underwent emergency surgery at our institute, the results of our study show that COVID-19 pneumonia is associated with poor liver function and coagulation function in acute abdomen patients with COVID-19 pneumonia. However, emergency surgery could not only improve the outcomes of COVID-19 pneumonia patients with acute abdomen, but also benefit the resolution of pulmonary inflammation.

COVID-19 might complicate the perioperative course of acute abdomen [13, 19]. The bulk of evidence revealed that SARS-CoV-2 RNA was identified in stool specimens [7, 20] and that the viral receptor angiotensin-converting enzyme 2 (ACE2) was highly expressed in gastrointestinal epithelial cells [21, 22], this evidence supported the conclusion that the digestive system is a potential target of SARS-CoV-2. In addition, infection-related biomarkers (including peripheral blood lymphocytes and WBCs) tend to decrease in patients with COVID-19 pneumonia [3, 4], while these indicators frequently increase in patients who only have acute abdomen. Blanco-Colino et al. also reported a case of suspected acute abdomen as an extrapulmonary manifestation of COVID-19 [19]. All of these results demonstrated that COVID-19 likely interferes with the accurate diagnosis and clinical assessment of acute abdomen.

To better carry out emergency surgery during the outbreak, our hospital has developed a detailed management strategy for acute abdomen patients. For patients with stable vital signs and local involvement (such as acute appendicitis alone, acute cholecystitis alone, and incomplete ileus) not requiring emergency surgery, conservative treatment in the outpatient department can be considered. If conservative treatment fails, emergency surgery should be performed immediately. The goal of emergency surgery is to remove the patient's lesions rapidly and effectively while minimizing the operation time and limiting the medical staff’s exposure.

The indications for emergency surgery should be strictly managed during the COVID-19 outbreak. The possible reasons for opposing surgical interventions for acute abdomen accompanied with COVID-19 pneumonia are as follows: 1) Surgical interventions on patients with COVID-19 may lead to contamination of the operating room and surgical equipment and risk transmission of the infection to healthcare providers and other patients in the hospital [17, 23]; 2) surgical treatment may trigger oxidative stress [24] and immunosuppression [25], which might hinder the clearance of SARS-CoV-2 and accelerate the progression of COVID-19 pneumonia. However, the scientific foundation of this theory is very weak. Jamali et al. reported that preoperative pneumonia only moderately increased the risk of mortality (OR= 1.2) in patients undergoing emergency surgery [14]. Moreover, an improvement of acute abdomen and pneumonia after surgery was observed in our study. A possible explanation for such results is that surgical treatment alleviated excessive inflammation and persistent immunosuppression caused by acute abdomen, which in turn contributed to clearance of the virus and resolution of lung inflammation. In addition, medical staff could effectively prevent SARS-CoV-2 infection through adherence to strict infection prevention and control protocols [16, 26]. No new infections were found in medical staff or patients throughout the hospitalization of patients with or without COVID-19 pneumonia in our study.

Current clinical observations have found that most COVID-19 patients have fever and acute abdomen patients often have fever. In our study, 5 patients (17.9%) presented with fever before emergency surgery. Some postoperative patients may present with fever, which may result from postoperative traumatic stress or residual abdominal infection. This makes it extremely difficult to identify the cause of fever and to identify COVID-19 in a timely manner. Elderly patients, especially those with pulmonary infections, are more susceptible to COVID-19 during the postoperative hospitalization period. Therefore, we monitored the patient's body temperature closely, and routine blood parameters, including PCT and CRP, were regularly retested. If necessary, a chest CT scan was performed again to monitor COVID-19 pneumonia progression. To ensure therapeutic efficacy, we streamlined treatments to reduce doctor-patient contact and avoid cross-infection.

This study had certain limitations that should be discussed. First, due to the lack of definite practical guidance for patients with both acute abdomen and COVID-19 pneumonia, the indication and timing of the surgical treatment was decided empirically instead of being based on evidence. Second, this was a small-sample nonrandomized retrospective study without strict inclusion and exclusion criteria, and as such, there were potential biases that could affect the comparison analysis. Third, the availability of clinical care and the diversity of COVID-19 management may limit the applicability of our results. However, to our knowledge, the results of our study provide the first evidence that emergency surgery could not only improve the outcomes of acute abdomen patients with COVID-19 pneumonia, but also benefit the resolution of pulmonary inflammation. These results hopefully lead to a consensus on the treatment and management of acute abdomen patients with or without COVID-19 during the COVID-19 outbreak.

## MATERIALS AND METHODS

### Study design and patient cohort

**Figure 6:**
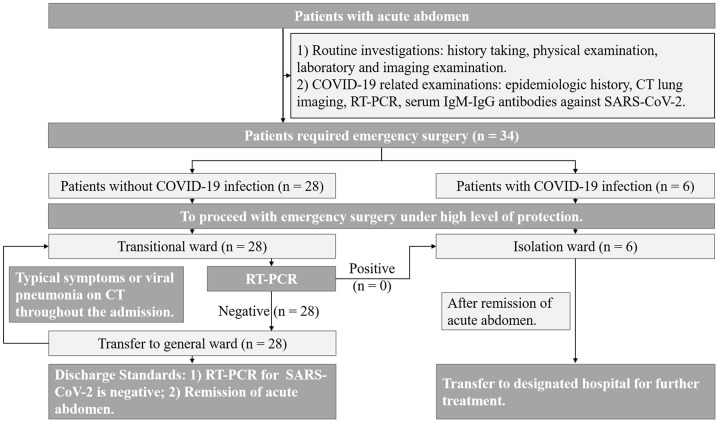
**Flow diagram for performing emergency surgery for acute abdomen patients during COVID-19 outbreak.**

We retrospectively reviewed 34 patients with acute abdomen who underwent emergency surgery from February 2, 2020, to March 18, 2020, at the Union Hospital affiliated with Tongji Medical College, Huazhong University of Science and Technology. This study protocol was approved by the ethics committee of our college. All patients signed an informed consent document indicating their understanding of the procedure and its potential complications as well as their approval to participate in the research study. A flow diagram of the emergency surgery protocol for patients with acute abdomen during COVID-19 outbreaks is presented in Figure 6.

### Preoperative work-up

After a detailed history and a complete physical examination, all patients with acute abdomen underwent routine laboratory testing (such as complete blood counts, serum biochemistry and tumor-marker screening) and imaging examination (such as chest X-ray, abdominal ultrasound, contrast-enhanced computed tomography (CT)). Prior to admission, all patients also completed a detailed risk assessment for COVID-19, including typical clinical manifestation and contact history with suspicious or confirmed COVID-19 patients within 14 days. CT lung imaging, quantitative reverse transcription-polymerase chain reaction (RT-PCR) and IgM-IgG antibodies against SARS-CoV-2 were also required for all patients to screen for potential infections. All suspected COVID-19 cases were treated as positive until confirmed. If emergency surgery was required, patients with positive indicators for infection must be taken directly to a designated COVID operation room through a predefined path. Due to the possible false negatives of test kits, all surgical procedures were carried out using a high level of protection, including masks, eye protection, gloves, caps and protective clothing, for the entire duration of the procedure.

### Postoperative work-up

All patients who were not excluded from possibly having COVID-19 were transferred to the isolation ward and transitional ward after surgery according to the status of the preoperative screening results. Medical staff were required to adhere to strict prevention and infection control protocols in addition to routine universal precautions, and all patients were advised to wear a mask throughout hospitalization. Patients in the transitional ward underwent another round of RT-PCR for SARS-CoV-2. If the screening yielded negative results, the patient was transferred and treated in a single room of the general ward for three to five days prior to transfer to a shared room. If patients presented with pyrexia of unknown origin, typical respiratory symptoms or CT imaging manifestations indicating viral pneumonia, they were transferred to the transitional ward to retest for SARS-CoV-2 infection by RT-PCR. If the screening yielded positive results, patients were transferred to the isolation ward for further treatment. After the remission of acute abdomen, patients in the isolation ward were advised to transfer to designated hospitals for the treatment of COVID-19.

### Data collection

Primary data, including clinical characteristics, the laboratory and imaging examination results, evidence of COVID-19, treatments, and clinical outcomes, were identified from medical reports. Blood samples from all participants were obtained through peripheral venipuncture. The following thresholds were considered the normal range of indicators: creatinine, 58-110 μmol/L; total bilirubin, 3-22 μmol/L; albumin, 35-50 g/L; alanine aminotransferase (ALT), 21-72 U/L; aspartate aminotransferase (AST), 17-59 U/L; hemoglobin (HGB), 130-175 g/L; white blood cell (WBC) count, (3.5-9.5)×10^9^/L; neutrophil count, (1.8-6.3)×10^9^/L; lymphocyte count, (1.1-3.2)×10^9^/L; C-reaction protein (CRP), < 8 mg/L; procalcitonin (PCT), < 0.5 μg/L; D-dimer, < 0.5 mg/L; activated partial thromboplastin time (APTT), 28-43.5 s; and prothrombin time (PT), 11-16 s. We adopted the classification system of the New Coronavirus Pneumonia Prevention and Control Program (7^th^ edition). According to this system, COVID-19 pneumonia cases were divided into four groups: mild, moderate, severe and critically ill. The discharge requirements for patients who only had acute abdomen include 1) remission of acute abdomen and 2) negative SARS-CoV-2 results by RT-PCR. However, for acute abdomen patients with COVID-19 pneumonia, obvious resolution of pulmonary inflammation and negative results by RT-PCR for two consecutive evaluation times were necessary for discharge.

### Statistical analysis

Statistical analysis was performed using SPSS 23.0 (SPSS Inc., Chicago, IL, USA), and diagrams of curves were drawn using Prism (version 6.0; GraphPad). Data are presented as numbers and percentages for categorical variables, and continuous data are expressed as the mean ± standard deviation (SD). Differences were considered significant at *p<0.05, **p <0.01 and ***p <0.001. ns: no significance.
